# TNF is a homoeostatic regulator of distinct epigenetically primed human osteoclast precursors

**DOI:** 10.1136/annrheumdis-2020-219262

**Published:** 2021-03-10

**Authors:** Cecilia Ansalone, John Cole, Sabarinadh Chilaka, Flavia Sunzini, Shatakshi Sood, Jamie Robertson, Stefan Siebert, Iain B McInnes, Carl S Goodyear

**Affiliations:** Institute of Infection, Immunity and Inflammation, University of Glasgow, Glasgow, UK

**Keywords:** cytokines, tumour necrosis factors, arthritis

## Abstract

**Objectives:**

Circulating myeloid precursors are responsible for post-natal osteoclast (OC) differentiation and skeletal health, although the exact human precursors have not been defined. Enhanced osteoclastogenesis contributes to joint destruction in rheumatoid arthritis (RA) and tumour necrosis factor (TNF) is a well-known pro-osteoclastogenic factor. Herein, we investigated the interplay between receptor activator of nuclear factor kappa-Β ligand (RANK-L), indispensable for fusion of myeloid precursors and the normal development of OCs, and TNF in directing the differentiation of diverse pre-OC populations derived from human peripheral blood.

**Methods:**

Flow cytometric cell sorting and analysis was used to assess the potential of myeloid populations to differentiate into OCs. Transcriptomic, epigenetic analysis, receptor expression and inhibitor experiments were used to unravel RANK-L and TNF signalling hierarchy.

**Results:**

TNF can act as a critical homoeostatic regulator of CD14^+^ monocyte (MO) differentiation into OCs by inhibiting osteoclastogenesis to favour macrophage development. In contrast, a distinct previously unidentified CD14^−^CD16^−^CD11c^+^ myeloid pre-OC population was exempt from this negative regulation. In healthy CD14^+^ MOs, TNF drove epigenetic modification of the RANK promoter via a TNFR1-IKKβ-dependent pathway and halted osteoclastogenesis. In a subset of patients with RA, CD14^+^ MOs exhibited an altered epigenetic state that resulted in dysregulated TNF-mediated OC homoeostasis.

**Conclusions:**

These findings fundamentally re-define the relationship between RANK-L and TNF. Moreover, they have identified a novel pool of human circulating non-MO OC precursors that unlike MOs are epigenetically preconditioned to ignore TNF-mediated signalling. In RA, this epigenetic preconditioning occurs in the MO compartment providing a pathological consequence of failure of this pathway.

**Key message**

**What is already known about this subject?**

- OC differentiation and maturation from myeloid precursors relies on receptor activator of nuclear factor kappa-Β (RANK) signalling and murine studies suggest that tumour necrosis factor (TNF) is a potent coactivator.
- One of the gold standard therapy for rheumatoid arthritis includes blocking TNF although a consistent portion of patients do not respond and display progressive joint destruction.

**What does this study add?**

- We have discovered a novel non-monocyte (MO) osteoclast (OC) precursor population in the human peripheral blood and demonstrated its epigenetic and transcriptomic imprint towards OC differentiation.
- We have demonstrated that TNF can act as a homoeostatic regulator of classical peripheral CD14^+^ MOs by inhibiting their differentiation into OCs to favour macrophages and showed how this mechanism is epigenetically perturbated in a portion of patients with rheumatoid arthritis (RA) with active disease.

**How might this impact on clinical practice or future developments?**

- These data provide an important insight into the cellular and epigenetic heterogeneity in RA and can be used to develop alternative therapeutics for those patients that do not respond to current therapies.

## Introduction

Osteoclasts (OCs) are polykarion bone-resorbing cells that can be derived from either yolk sac erythro-myeloid progenitors or bone marrow/circulating monocyte (MO) precursors^1^ supported by the receptor activator of nuclear factor kappa-Β (RANK) receptor and tumour necrosis factor (TNF) family cytokine RANK-ligand (RANK-L).^2 3^ Circulating MO also have the capacity to differentiate into macrophages (Mϕ) or dendritic cells (DCs).^4 5^ Recently, human ontogenetic and transcriptomic studies have re-classified DCs, MO, MO-derived cells and tissue resident Mϕ based on their differentiation from specific precursor cells.^6 7^ While murine studies have identified specific OC myeloid precursors^8^ that play a crucial role in post-natal OC differentiation in vivo,^1^, it is unclear whether specific non-MO circulating OC precursors exist in humans. Moreover, how such precursors respond to competing differentiating and activating cytokine signals and select their eventual cell fate is poorly understood.

Murine studies have shown that in combination with RANK-L, TNF is a direct maturation/activation stimulus of OC precursors.^9–11^ It has been assumed that TNF mediates equivalent actions across human OC precursors. Consistent with this notion, TNF is one of the main mediators of joint inflammation in inflammatory diseases such as rheumatoid arthritis (RA), which is associated with articular damage and systemic bone loss.^12 13^ Furthermore, in the clinical setting, treatment with TNF inhibitors has been shown to reduce articular erosion.^14^ However, the precise effects of TNF acting directly on human myeloid/MO/OC precursors remains ill-defined. Inflamed tissues, both in acute or chronic states, exhibit elevated levels of TNF, usually associated with MO recruitment and maturation even in the presence of RANK-L.^15^ It is unclear why in environments that are favourable to osteoclastogenesis, a preponderance of infiltrating myeloid precursors does not differentiate down the OC lineage.

Herein, we report the existence of a human CD14^−^CD16^−^CD11c^+^ myeloid precursor population that is epigenetically predisposed to rapidly differentiate into OCs. Notably, this population is unresponsive to a previously unrecognised homoeostatic TNF-mediated signal that fundamentally governs cell fate decisions in CD14^+^ MOs that favours Mϕ development. Moreover, we provide evidence that this effect is mediated by TNF via epigenetic regulation of the RANK promoter in circulating CD14^+^ MOs and that this pathway is perturbed in RA.

## Materials and methods

Detailed experimental procedures and analyses are provided in online supplemental files.

10.1136/annrheumdis-2020-219262.supp1 Supplementary data

## Results

### Distinct epigenetically primed OC precursors in human blood

**Figure 1:**
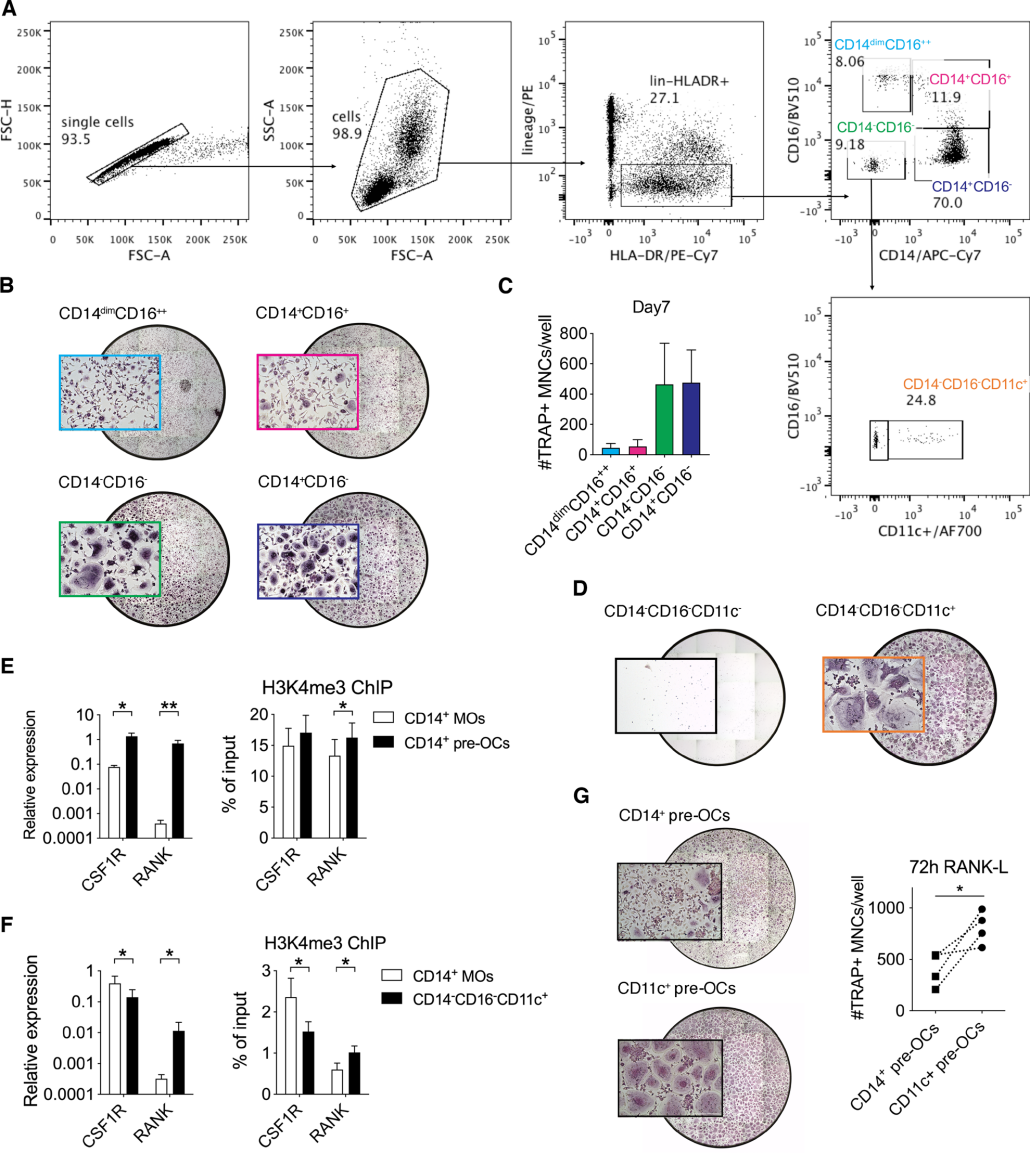
Identification and epigenetic state of osteoclast (OC) precursor populations in human blood. (A–D) Sorted peripheral blood mononuclear cells (PBMCs) were plated overnight with 25 ng/mL macrophage-colony stimulating factor (M-CSF) and subsequently 25 ng/mL receptor activator of nuclear factorkappa-B ligand (RANK-L) was added to differentiate cells into OCs. At day 7 cells were fixed and stained for TRAP. (A) Representative flow cytometric plots showing the gating strategy used to sort monocyte (MO) subsets and CD14^−^CD16^−^CD11c^+^ myeloid cells. Cells were pre-gated as single Lin^−^ (CD19^−^CD3^−^CD15^−^CD56^−^) HLA-DR^+^. Numbers indicate percentage of parental subset. (B) Representative digital reconstructed wells at 4× (with a boxed 10× magnification) and (C) quantification of multinucleated (nuclei ≥3) cells (MNCs) TRAP+ (purple) OC differentiated from CD14^dim^CD16^++^, CD14^+^CD16^+^, CD14^+^CD16^−^ and CD14^−^CD16^−^ subsets. (D) Representative digital images of TRAP stained wells at 4× with boxed 10× magnification images of CD14^−^CD16^−^CD11c^−^ and CD14^−^CD16^−^CD11c^+^ cells. (E–G) CD14^+^ MOs and CD14^−^CD16^−^CD11c^+^ precursors were magnetically enriched from PBMCs (purity ≥96%) and incubated overnight with 25 ng/mL M-CSF to generate pre-OCs. (E–F) CSF1R and RANK mRNA expression (left hand side) and ChIP assay for H3K4me3 at promoter regions (right-hand side) of CSF1R and RANK genes in either (E) CD14^+^ MOs before and after 25 ng/mL M-CSF overnight stimulation (CD14^+^ pre-OCs) and (F) in freshly isolated donor-matched CD14^+^ MOS and CD14^−^CD16^−^CD11c^+^ myeloid cells. Bars show mean±SD. Data were analysed with two-way analysis of variance (ANOVA) for paired data and Sidak’s multiple comparisons test or paired Wilcoxon t-test (n=3–4 in E and n=6 in F). *p≤0.05; **p≤0.01. (G) Representative image of TRAP staining and quantification of TRAP+ MNCs/well of CD14^+^ pre-OCs and CD11c^+^ pre-OCs after 72 hours of 25 ng/mL RANK-L. Dotted lines indicate donor-matched samples. Data were analysed with Mann-Whitney test (n=4). *p≤0.05.

Circulating CD14^+^ MOs are regarded as classical OC precursors,^16^ but the existence of a distinct circulating human precursor is not known. To investigate the osteoclastogenic potential of circulating human myeloid cells we used fluorescence-activated cell sorting (figure 1A) to purify circulating MO subsets (classical Lin^−^HLA^−^DR^+^CD14^+^CD16^−^, intermediate Lin^−^HLA^−^DR^+^CD14^+^CD16^+^ and non-classical Lin^−^HLA^−^DR^+^CD14^dim^CD16^++^) and other myeloid cells (Lin^−^HLA^−^DR^+^CD14^−^CD16^−^). Culture of these populations with macrophage-colony stimulating factor (M-CSF) and RANK-L verified that classical MOs differentiate into mature OCs while intermediate and non-classical populations only produced few and variable numbers of small OCs^17^ (figure 1B, C). Intriguingly, purified Lin^−^HLA^−^DR^+^CD14^−^CD16^−^ myeloid cells differentiated into OCs, with numbers comparable to classical CD14^+^ MOs (figure 1B, C). Lin^−^HLA^−^DR^+^CD14^−^CD16^−^ myeloid cells can be subdivided via CD11c expression into populations that contain conventional pre-DCs (CD14^−^CD16^−^CD11c^+^) and plasmacytoid DCs (CD14^−^CD16^−^CD11c^−^)^7^; only the CD14^−^CD16^−^CD11c^+^ population was able to adhere and differentiate into mature OCs (figure 1D).

The in vivo kinetics of osteoclastogenesis from precursors to a mature OC are not clear in humans, however, murine studies have shown that preconditioned cells can mature into OCs within 48 hours.^18^ The in vitro generation of a mature OCs from circulating CD14^+^ MOs or in vitro differentiated MO-derived pre/immature DCs or Mϕ takes in excess of 7–14 days.^5 19 20^ We hypothesised that this reflects the non-preconditioned state of the precursor and thus time is required for in vitro cell differentiation/trans-differentiation. In support of this, CD14^+^ MOs are preincubated overnight with M-CSF to generate CD14^+^ pre-OCs. This preincubation leads to upregulation of RANK and CSF1R (M-CSF receptor) transcripts (figure 1E),^21^ and increased tri-methylation of histone H3 at lysine 4 (H3K4me3) in the RANK promoter (figure 1E), indicative of a transcriptionally active epigenetic state.^22^ In comparison, immediate ex vivo evaluation of the identified CD14^−^CD16^−^CD11c^+^ myeloid population revealed that they have higher levels of RANK transcript and decreased levels of CSF1R compared with classical CD14^+^ MOs (figure 1F). This was associated with an increased level of H3K4me3 at the RANK promoter but less at the CSF1R promoter of CD14^−^CD16^−^CD11c^+^ myeloid population compared with donor-matched CD14^+^ MOs (figure 1F). Pre-OCs from both CD14^+^ MOs and CD14^−^CD16^−^CD11c^+^ cells were generated by M-CSF overnight incubation. Assessment of OC differentiation 72 hours post-RANK-L stimulation, revealed that while CD14^+^ pre-OCs produce few small OCs at this time point, CD11c^+^ pre-OCs rapidly differentiate into macroscopically larger OCs, with numbers significantly higher than those differentiated from donor-matched CD14^+^ pre-OCs (figure 1G). Taken together, these results suggest that the circulating CD14^−^CD16^−^CD11c^+^ myeloid population contains OC precursors that are in an epigenetically precommitted state to rapidly differentiate into mature OCs.

### TNF over-rides RANKL-driven osteoclastogenesis of CD14^+^ circulating precursors but not CD14^−^CD16^−^CD11c^+^ precursors

**Figure 2:**
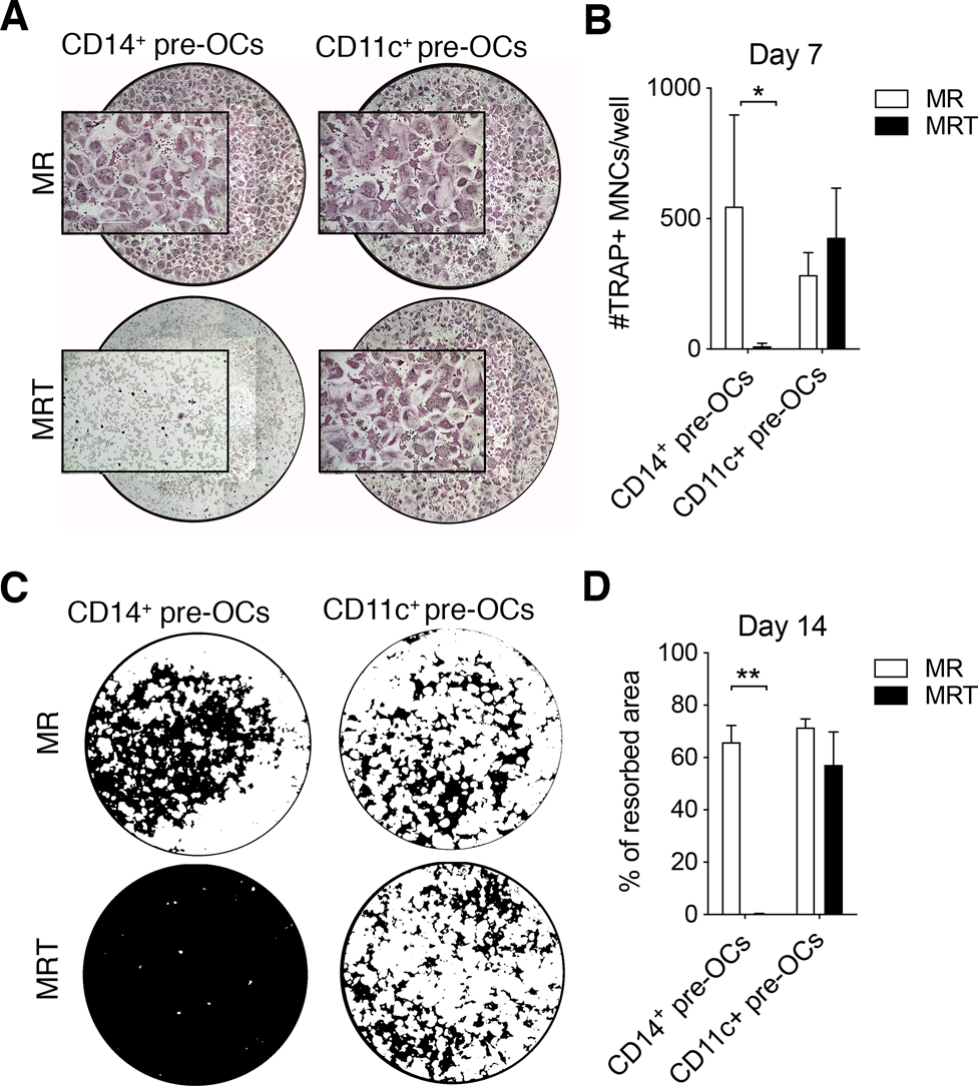
Tumour necrosis factor (TNF) over-rides receptor activator of nuclear factorkappa-B ligand (RANKL)-driven osteoclastogenesis of CD14^+^ pre-osteoclasts (OCs) but not CD11c^+^ pre-OCs. (A–B) Freshly isolated peripheral blood mononuclear cells (PBMCs) were stained for flow cytometry and CD16^−^CD14^+^ monocytes (MOs) and CD14^−^CD16^−^CD11c^+^ precursors were sorted, incubated overnight with 25ng/mL macrophage-colony stimulating factor to generate pre-OCs, and then differentiated into OCs for 7 days with 25 ng/mL RANK-L (MR) ±10 ng/mL TNF (MRT). (A) Representative digital reconstructed TRAP stained wells at 4× with boxed 10× magnification and (B) quantification of numbers of TRAP+ multinucleated cells (MNCs) per well. Data were analysed with two-way analysis of variance (ANOVA) and Holm-Sidak’s multiple comparisons test for paired data (n=3). *p≤0.05. Error bars show mean±SD of n=3. (C–D) CD14^+^ MOs and CD14^−^CD16^−^CD11c^+^ precursors were magnetically enriched, seeded onto mineral-coated wells, and differentiated into OCs for 14 days as in (A, B). (C) Digital images of resorption pits (mineral substrate in black; resorption pits in white) and (D) % of resorption. Statistical analysis was performed using two-way ANOVA and Sidak’s multiple comparisons test for paired data. Error bars=mean±SD of n=3. **p≤0.01.

TNF is a well-known pro-osteoclastogenic factor, both in vitro^9 10 23^ and in vivo.^24–26^ However, nothing is known about how human circulating precursors respond to simultaneous exposure to TNF family member cytokines, as would be expected in a physiological setting. Therefore, we simultaneously stimulated human CD14^+^ pre-OCs and CD11c^+^ pre-OCs with RANK-L and TNF. Unexpectedly, we found that synchronised stimulation of CD14^+^ pre-OCs with RANK-L and TNF resulted in substantial inhibition of osteoclastogenesis (figure 2A, B). The resulting cells exhibited Mϕ morphology, although dissimilar to either regulatory M-CSF-driven Mϕ (M-Mϕ) or pro-inflammatory GM-CSF-driven Mϕ (GM-Mϕ) (online supplemental figure S1).

In comparison to what was observed in CD14^+^ pre-OCs, TNF was unable to inhibit the generation of mature OCs derived from CD11c^+^ pre-OCs (figure 2A, B). Extending the duration of differentiation to 14 days, we found that TNF completely abrogated the resorptive activity of CD14^+^ pre-OCs but had no effect on CD11c^+^ pre-OCs resorption (figure 2C, D). In order to demonstrate that CD11c^+^ pre-OCs were not completely unresponsive to TNF, the secretion of relevant cytokines was evaluated. Notably, TNF stimulation resulted in the increased secretion of interferon-γ in CD11c^+^ pre-OCs but not CD14^+^ pre-OCs (online supplemental figure S2). Conversely, TNF stimulation resulted in the secretion of interleukin (IL)-12, IL-1β and IL-6 in CD14^+^ pre-OCs but not CD11c^+^ pre-OCs (online supplemental figure S2). Thus, circulating CD14^−^CD16^−^CD11c^+^ precursors and CD14^+^ MOs display a fundamental difference in their response to TNF, with CD14^−^CD16^−^CD11c^+^ precursors able to differentiate into mature and functional OCs.

### TNF-mediated epigenetic modification of the RANK promoter controls RANK transcription and expression in CD14^+^-derived OC precursors

**Figure 3:**
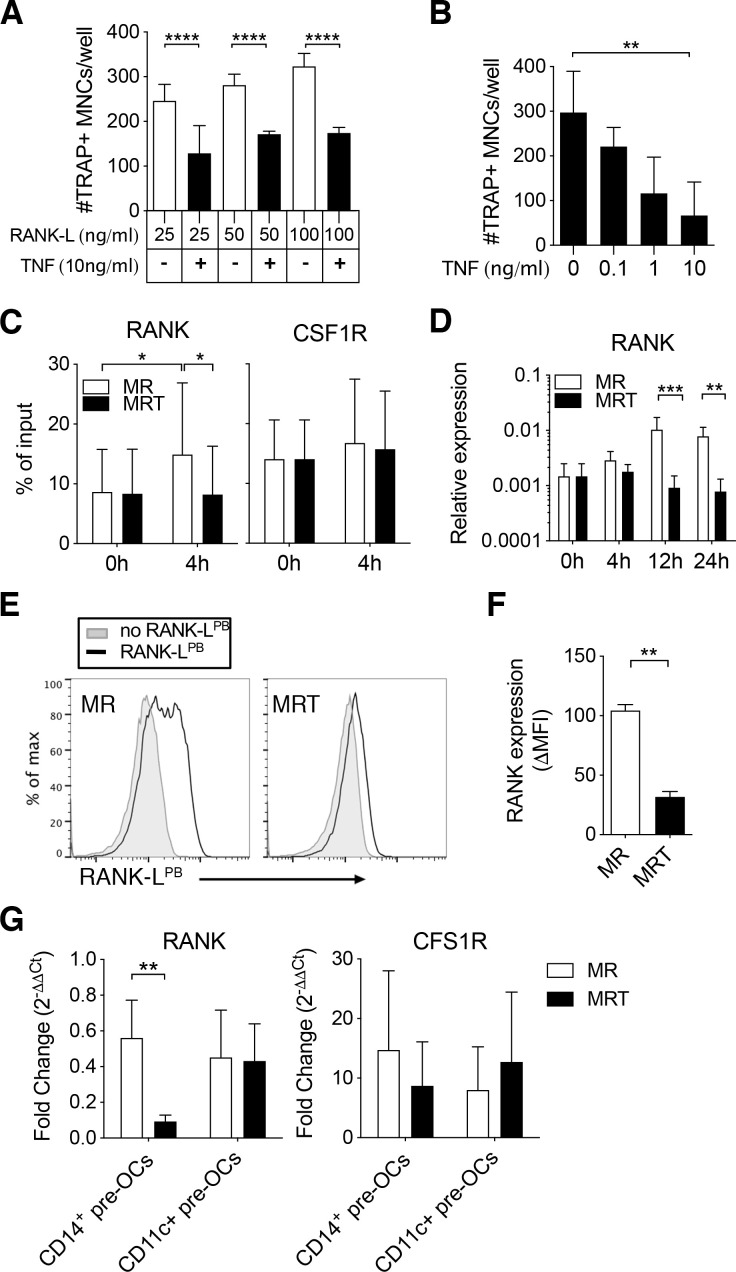
Tumour necrosis factor (TNF) mediates epigenetic modification of the receptor activator of nuclear factor kappa-Β (RANK) promoter and controls RANK transcription and expression in CD14^+^ pre-osteoclasts (OCs). (A–F) Enriched CD14^+^ monocytes (MOs) were incubated overnight with 25 ng/mL to generate CD14^+^ pre-OCs. OCs were differentiated in the presence of RANK-ligand (RANK-L)±TNF. (A) Quantification of number of TRAP+ multinucleated cells (MNCs)/well after 6 days of culture in increasing concentrations of RANK-L (25, 50 or 100 ng/mL)±10 ng/mL TNF. Error bars show mean±SD of n=3. Data were analysed with two-way analysis of variance (ANOVA) and Tukey’s multiple comparisons test. ****p≤0.0001. (B) Quantification of TRAP+ MNCs/well after 6 days of 25 ng/mL RANK-L±TNF at different concentrations (0.1, 1 or 10 ng/mL). Bars=mean±SD of n=3. Data were analysed with Friedman test with Dunn’s multiple comparisons test, comparing to 0 ng/mL TNF. **p≤0.01. (C–F) CD14^+^ pre-OCs were differentiated in the presence of 25 ng/mL macrophage-colony stimulating factor (M-CSF)+25 ng/mL RANK-L (MR) or MR +10 ng/mL TNF (MRT). (C) ChIP assay for H3K4me3 at the RANK promoter (left-hand side) and at the CSF1R promoter (right-hand side) on CD14^+^pre-OCs (0 hour) and after 4 hours MR or MRT stimulation. Bars show mean±SD of n=4. Data were analysed with two-way ANOVA for paired data and Sidak’s multiple comparisons test. *p≤0.05. (D) mRNA expression of RANK was evaluated at 0, 4, 12, and 24 hours after cytokine addition on CD14^+^ pre-OCs. Data were analysed with two-way ANOVA and Dunnet’s multiple comparisons test. **p≤0.01; ***p<0.001; n=4. (E) Representative histograms of uptake of fluorescent RANK-L (RANK-L^PB^; black lines) after 72 hours of MR or MRT stimulation. Grey filled lines indicate unstained controls. (F) Graph shows quantification of RANK-L^PB^ uptake; ΔMFI was calculated by subtraction of the unstained background fluorescence. Statistical significance was assessed via one-way ANOVA and Holm-Sidak’s multiple comparisons test. **p≤0.01; n=3. (G) CD14^+^ pre-OCs and CD11c^+^ pre-OCs were stimulated with MR or MRT for 4 hours. Graphs show RANK and CSF1R mRNA fold change, calculated by normalising to values at 0 hour prior stimulation (on pre-OCs after overnight M-CSF). Statistical significance was calculated using 2-way ANOVA for paired data and Sidak’s multiple comparisons test. **p≤0.01 *p≤0.05. Error bars show mean±SD of n=5.

To further explore the unexpected inhibition of osteoclastogenesis by TNF, we extended our cellular and molecular characterisation of CD14^+^ pre-OCs. Increasing RANK-L concentration to potentially outcompete the simultaneous TNF signal did not restore OC differentiation (figure 3A). In comparison, there was a TNF dose-dependent reduction in the development of mature multinucleated OCs (figure 3B). To demonstrate TNF specificity and exclude the possibility of cross-contamination, addition of the TNF inhibitor etanercept^27^ reversed osteoclastogenesis (online supplemental figure S3A, B).

To characterise the time-dependence of this effect of TNF, CD14^+^ pre-OCs were stimulated with suboptimal levels of RANK-L that resulted in the appearance of mono-nucleated and bi-nucleated TRAP^+^ cells after 72 hours stimulation. The addition of TNF at this 72 hours time-point did not inhibit osteoclastogenesis (online supplemental figure S4A, B) but rather caused an increase in OCs (online supplemental figure S4C), consistent with many previous studies.^28^ Therefore, the kinetics of exposure to TNF are critical for its effect on differentiation of CD14^+^ pre-OCs into mature OCs; with precursors initially having to commit to the OC lineage before there is a positive synergy between RANK and TNF.

To investigate the molecular mechanism responsible for early TNF exposure-mediated inhibition observed in CD14^+^ pre-OCs, we examined the epigenetic state of the RANK promoter and the resulting transcript and protein expression. On 4 hours stimulation with RANK-L, CD14^+^ pre-OCs displayed enhanced H3K4me3 at the RANK promoter, with subsequent increased RANK transcript at 12 hours and 24 hours (figure 3C, D). The simultaneous addition of TNF with RANK-L suppressed this enhanced H3K4me3 and upregulation of transcript (figure 3C, D). This correlated with suppression of RANK expression at the cell surface (figure 3E, F). Notably, H3K4me3 levels at the CSF1R promoter and CSF1R transcripts were unaltered on RANK-L ±TNF stimulation (figure 3C and online supplemental figure S5). As noted before, in contrast to CD14^+^ pre-OCs, CD11c^+^ pre-OCs were not sensitive to the TNF inhibition (figure 2). To investigate the disconnect between the two cell types, we evaluated the transcriptional expression of RANK post-TNF treatment in donor-matched CD14^+^ pre-OCs and CD11c^+^ pre-OCs. Simultaneous addition of TNF with RANK-L suppressed the level of RANK in CD14^+^ pre-OCs but not in CD11c^+^ pre-OCs (figure 3G). Combined, these data suggest that TNF-mediated signalling in CD14^+^-derived OC precursors, but not CD14^−^CD16^−^CD11c^+^ precursors, epigenetically modulates the RANK locus, resulting in loss of transcript and protein expression.

### TNF over-rides RANKL-driven osteoclastogenesis of CD14^+^ circulating precursors via a TNFR1 and canonical NF-κB pathway

**Figure 4:**
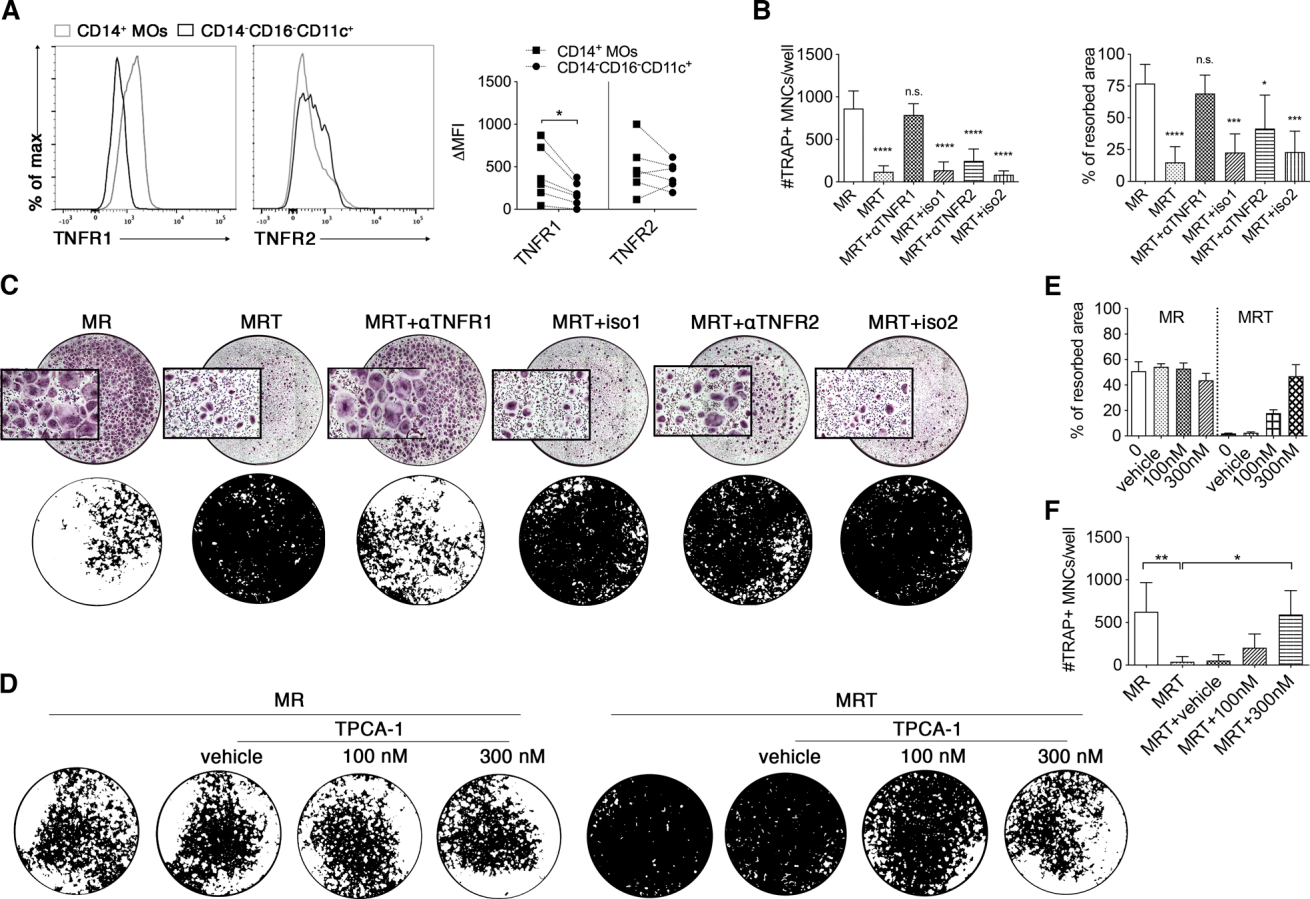
Tumour necrosis factor (TNF) over-rides receptor activator of nuclear factorkappa-Β ligand (RANKL)-driven osteoclastogenesis of CD14^+^ pre-osteoclasts (OCs) via a TNFR1 and canonical NF-kB pathway. (A) Freshly isolated peripheral blood mononuclear cells (PBMCs) were stained for flow cytometry. Representative histograms showing TNFR1 and TNFR2 expression on CD14^+^ monocytes (MOs) and CD14^−^CD16^−^CD11c^+^ precursors and quantification of Δmean fluorescence intensity (MFIs) (calculated by subtracting the MFI of the TNFR to the relative MFI of the isotype control). Cells were pre-gated on single Lin^−^(CD3^−^CD19^−^CD15^−^CD56^−^) HLA-DR^+^. Dotted lines indicate donor-matched samples. Data were analysed with Wilcoxon matched-paired signed rank test (n=6). *p≤0.05. (B–F) CD14^+^ pre-OCs were differentiated in the presence of 25 ng/mL macrophage-colony stimulating factor+25 ng/mL RANK-L (MR) or MR +10 ng/mL TNF (MRT). (B) Quantification of numbers of TRAP+ MNCs/well (top graph) and % of resorbed area (bottom graph) calculated, respectively at day 7 and day 10 of MR or MRT cultures±antibody blocking TNFR1 or TNFR2 (αTNFR1 and αTNFR2) or±the respective isotype controls (iso1 and iso2, respectively). Statistical significance was assessed with two-way analysis of variance (ANOVA) and Sidak’s multiple comparisons test, comparing all data to MR. Error bars=mean±SD of n=3. ***p<0.001, ****p<0.0001. (C) Representative digital reconstructed wells of TRAP staining and 10× magnifications (in purple) at day 7 (top) and mineral-coated wells (mineral substrate in black; resorption pits in white) at day 10 (bottom) of CD14^+^-derived OC culture. (D) Representative digital images of resorption assay at day 10, (E) quantification of % of resorbed area and (F) quantification of numbers of TRAP+ MNCs/well differentiated at day 7 with MR or MRT in the presence of an IKK-β inhibitor at 100 nM (MRT+100 nM) or 300 nM (MRT+300 nM) or+vehicle control (MRT+v). (E) Mean±SD of one representative experiment of n=4. Data in (F) were analysed with Friedman analysis of variance and Dunn’s multiple comparisons test. *p≤0.05; **p≤0.01. Error bars indicate mean±SD of n=3.

RANK-L and TNF belong to the same TNF superfamily; stimulation of their respective receptors results in activation of NF-κB. However, TNF stimulation of TNFR1 and TNFR2 primarily leads to activation of the canonical and non-canonical NF-κB pathways, respectively.^29 30^ RANK-L stimulation of RANK predominately leads to the activation of the non-canonical NF-κB pathway.^31^ TNFR1 and TNFR2 are expressed on both CD14^+^ circulating MOs and CD14^−^CD16^−^CD11c^+^ myeloid cells (figure 4A).^32^ However, the level of TNFR1 is significantly lower on CD14^−^CD16^−^CD11c^+^ precursors (figure 4A). To assess the role of TNFR1 and TNFR2 in the TNF-mediated inhibition of OC differentiation from CD14^+^ MOs, we utilised TNFR1-specific and TNFR2-specific blocking antibodies. Blockade of TNFR1, but not TNFR2, mitigated the inhibitory effect of TNF on OC numbers and function (figure 4B, C). Given that TNFR1-mediated signalling is through the canonical NF-κB pathway (via the IκB kinase (IKK) complex formed by IKK-α, IKK-β and NEMO),^30^ we used a selective IKK-β inhibitor (TPCA-1) to specifically block this signalling cascade. Inhibition of IKK-β significantly suppressed the ability of TNF to inhibit OC differentiation and activation (figure 4D–F). Importantly, TPCA-1 did not affect RANK-L-induced osteoclastogenesis or their resorptive capacity (figure 4D, E), as this is primarily driven by non-canonical NF-κB signalling.^33–35^ TNFR1-mediated signals have also been associated with initiation of caspase cascades and subsequent apoptosis. TNF stimulation of CD14^+^ pre-OCs resulted in decreased caspase activation and no change in apoptosis levels (online supplemental figure S6). Interestingly, RANK-L-mediated differentiation of CD14^+^ pre-OCs for 72 hours resulted in upregulation of TNFR2 (online supplemental figure S7A, B). This corresponded with TNFR2-dependent TNF-mediated enhancement of OC differentiation in these precommitted OC precursors (online supplemental figure S7C, D). Taken together, our data reveal a fundamental dual role of TNF in enhancing or blocking OC differentiation. Early exposure to concomitant RANK-L and TNF activates the canonical NF-κB pathway via TNFR1 and halts osteoclastogenesis; in contrast, later TNF addition on precommitted pre-OCs aids RANK-L and osteoclastogenesis via TNFR2 and non-canonical NF-κB signalling. This may also partly explain why circulating CD14^−^CD16^−^CD11c^+^ precursors are unresponsive to TNF-mediated inhibition, given their lower levels of TNFR1 (figure 4A).

### Patients with RA have a perturbed myeloid compartment and TNF does not negatively regulate OC differentiation

**Figure 5:**
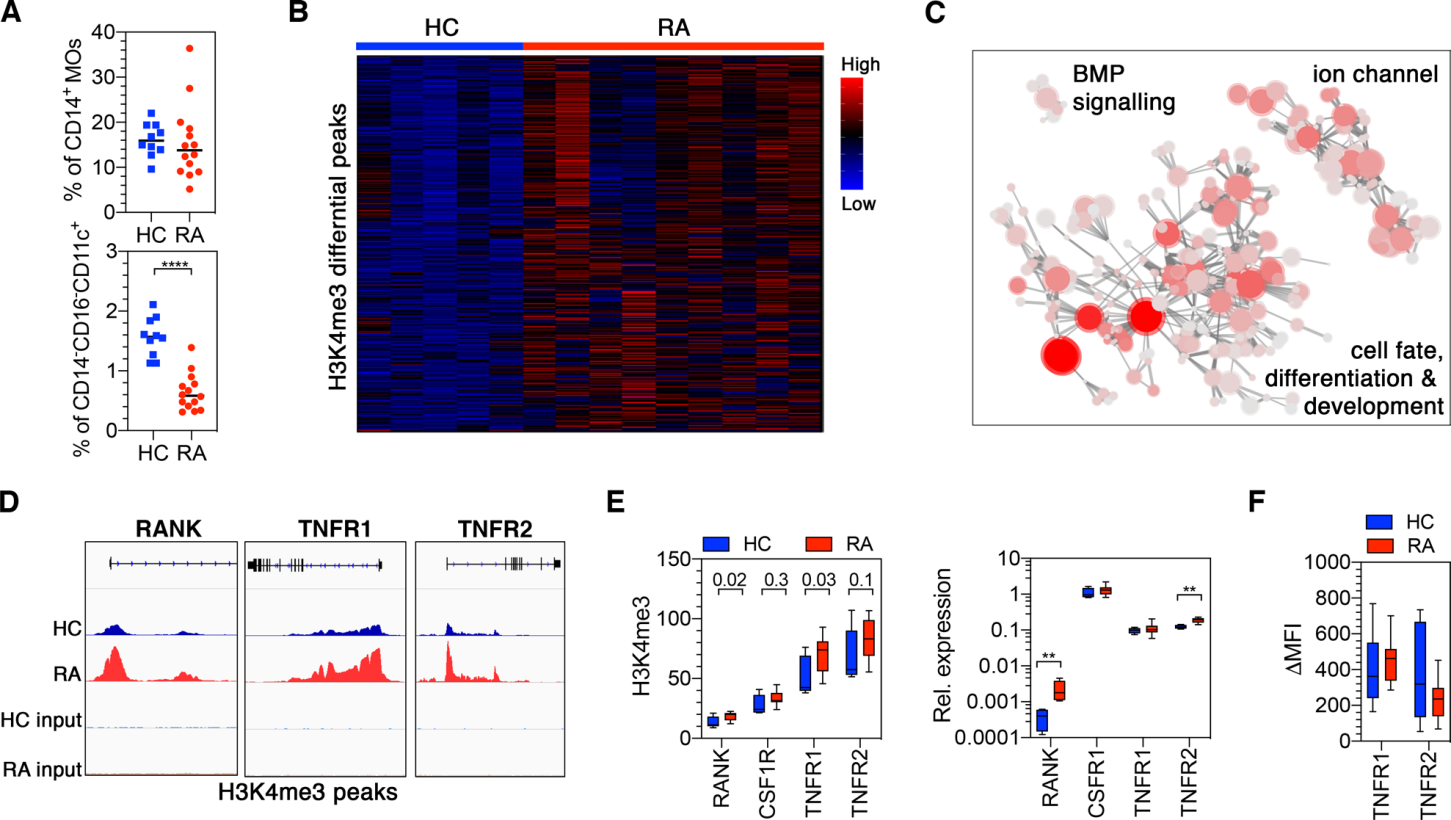
Patients with rheumatoid arthritis (RA) have epigenetically perturbed CD14^+^ monocytes with increased receptor activator of nuclear factor kappa-Β (RANK) expression. (A) Freshly isolated peripheral blood mononuclear cells (PBMCs) from healthy controls (HC) and RA were stained for flow cytometry and percentages of CD14^+^ MOs and CD14^−^CD16^−^CD11c^+^ precursors in PBMCs were calculated. Statistical significance was calculated using unpaired t*-*test (n=10 HC and n=14 RA). ****p≤0.0001. (B) Diffbind normalised peak intensity heatmap highlighting the size and consistency of differences between HC and RA. Samples are given by column and the 6763 significantly differential peaks (<5% FDR) by row. Colour intensity indicates row scaled (z-score) peak intensity with blue as low and red as high. Rows have been hierarchically clustered using Spearman distances. (C) Network plot of the enriched (p<0.0001) Gene Ontologies for the 6763 significantly differential peaks (FDR<5%). Nodes denote Gene Ontologies and edges join nodes where >50% significant peak containing genes are shared. Node colour intensity represents enrichment (−log10p) and node size the number of significant peaks containing genes. Representative names for node clusters are given. (D) Genome Browser traces of the RANK, TNFR1 and TNFR2 H3K4me3 promoter peaks. (E) Input normalised peak intensity boxplots for the RANK, TNFR1 and TNFR2 promoter peaks (top) and relative mRNA expression measured on freshly isolated CD14^+^ monocytes (MOs) from HC and RA (bottom). Unadjusted diffbind p values are given in the top graph. Two-way analysis of variance (ANOVA) and Sidak’s multiple comparisons test was used for the analysis in the bottom graph. Graph shows box and whiskers with min to max of n=4 for HC and n=11 for RA. *p≤0.05; **p≤0.01. (F) Freshly isolated PBMCs from HC and RA were stained for flow cytometry to evaluate TNFR1 and TNFR2 expression. Box and whiskers show TNFR1 and TNFR2 expression on CD14^+^ MOs in Δmean fluorescence intensity (MFIs) for n=13 each group. Error bars shows min to max variation.

Cell in the myeloid compartment and TNF play key roles in RA pathology. Analysis of circulating CD14^+^ MOs and CD14^−^CD16^−^CD11c^+^ precursors in our RA cohort showed that while the abundance of CD14^+^ MOs was not affected in RA, CD14^−^CD16^−^CD11c^+^ precursors were significantly reduced (figure 5A). This is consistent with previous studies showing reduced mDCs in both RA bloodstream and inflamed synovium.^36^ Whether this is due to their rapid recruitment and differentiation in situ or to their minor contribution to bone erosion in RA pathology is not known. However, recent independent studies both in the blood and the synovium have interrogated the molecular signature of both CD14^+^ MOs and CD1C pre-DC (which are the closest subset to our CD11c^+^ myeloid precursors) in RA.^37 38^ Analysis of this publicly available data revealed a high transcriptional correlation between blood and synovial CD1C cells (online supplemental figure S8A), indicating a common cell lineage origin. Notably, among the most differentially expressed gene, RANK was dramatically upregulated in the CD1C synovial counterpart, compared with circulating cells (online supplemental figure S8B). These data taken together suggest that CD11c^+^ (or CD1C cells) are present in the inflamed synovium and have the molecular potential to differentiate into OC; although their contribution to RA bone erosion remains elusive.

In contrast, although the abundance of circulating MOs was unaffected in RA, prior studies have shown that their transcriptional profile is different from non-inflammatory controls.^39^ We therefore hypothesised, having shown that the epigenetic status of CD14^+^ MOs controls the inhibitory TNF effect, that RA blood CD14^+^ MOs may exhibit a distinct epigenetic state. To gain further insight into this dysregulation, genome-wide H3K4me3 histone modification was investigated using ChIPseq. Comparison between patients with biological naïve RA with moderate to severe disease (DAS28=4.44±0.9) and healthy control CD14^+^ MOs, identified 6764 significantly differential peaks (adjusted p>0.05) (figure 5B). Pathway enrichment and network analysis revealed that these differential peaks of RA MOs were primary associated with cell differentiation and development pathways (figure 5C and online supplemental dataset 1).

10.1136/annrheumdis-2020-219262.supp2 Supplementary data

Based on the observed changes in RANK, CSF1R, TNFR1 and TNFR2 in healthy MOs, a focused analysis of the ChIPseq data showed that there was a significant increase of H3K4me3 in RA CD14^+^ MOs at the RANK and TNFR1 loci but not TNFR2 and CSF1R (figure 5D, E). This corresponded with an increased level of RANK transcript but not TNFR1 (figure 5E). However, increased TNFR2 transcript was detected in RA CD14^+^ MOs (figure 5E), although this did not correspond to a significant increase in cell surface expression (figure 5F).

**Figure 6:**
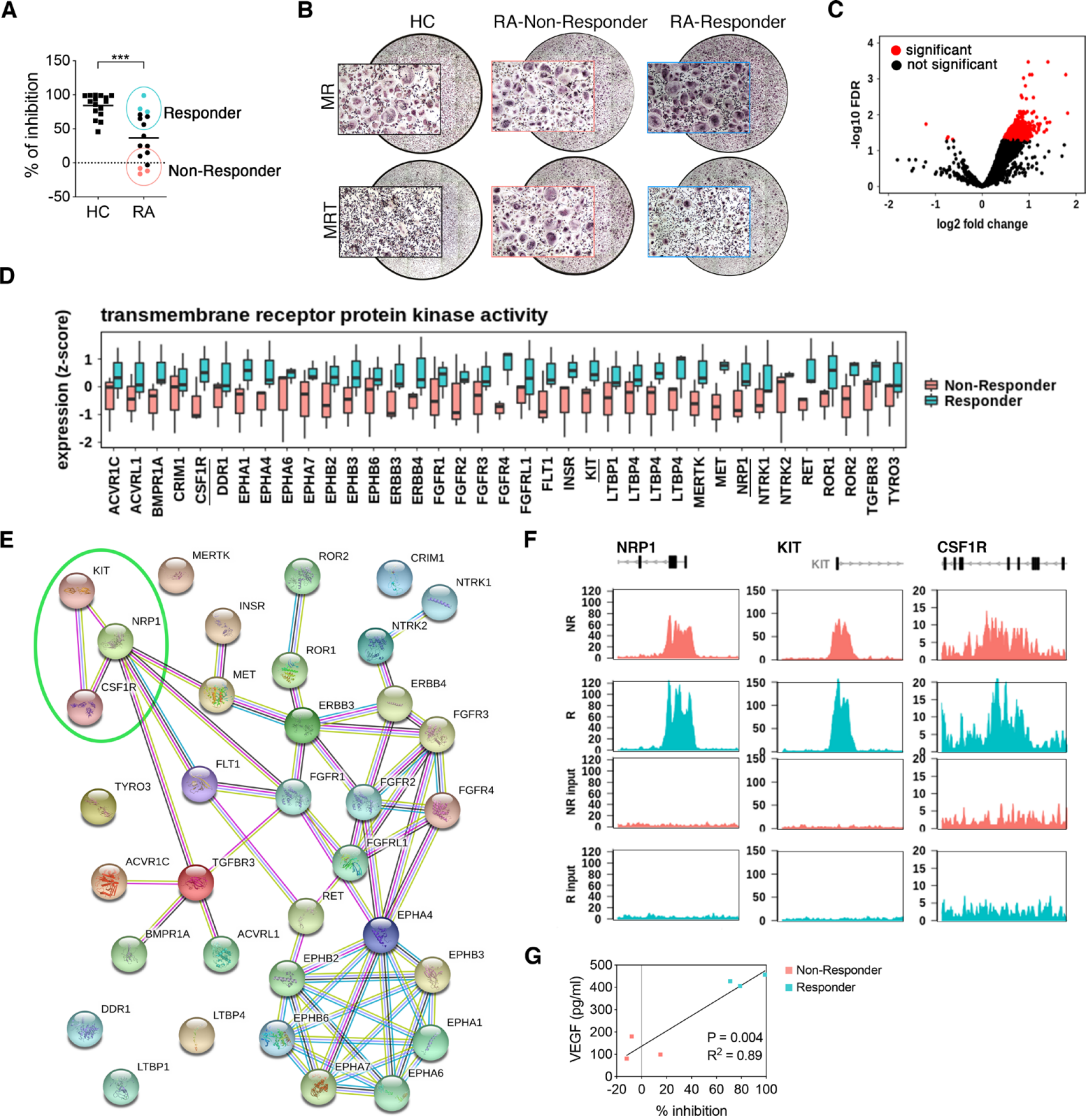
Tumour necrosis factor (TNF) does not inhibit CD14^+^-derived osteoclast (OC) differentiation in a portion of patients with rheumatoid arthritis (RA). (A–B) CD14^+^ pre-OCs were differentiated for 7 days into OCs in the presence of 25 ng/mL macrophage-colony stimulating factor (M-CSF) and receptor activator of nuclear factorkappa-Β ligand (RANK-L) (MR) ±10 ng/mL TNF (MRT). (A) Percentage of inhibition of OC differentiation was calculated as 100−[(100×#OCs_(MRT)_)/#OCs_(MR)_] in patients with healthy controls (HC) and RA. Light red dots indicate non-responder group and light blue dots the responder using in subsequent ChIP analysis. Data were analysed with Mann Whitney test (n=16). **p≤0.01. (B) Representative reconstructed wells and 10× magnifications of TRAP staining of one representative HC, one RA non-responder and one RA responder, in MR and MRT conditions. OCs=TRAP+ multinucleated cells, MR=M-CSF+RANK L, MRT=M-CSF+RANK-L+TNF. (C) Volcano plot for RA responders versus non-responders across the 65 717 consensus peaks. Significantly differential peaks (<5% FDR) are highlighted in red. Positive fold changes denote greater peak intensity in RA responders. (D) Boxplot of the 35 significantly differential peaks (<5% FDR) between RA non-responders and RA responders that are associated with the top enriched (p*<*0.0001) gene ontology transmembrane receptor protein kinase activity. Individual peaks (and the gene they are associated with) are given on the x-axis and the Diffbind normalised peak intensity (per peak z-score) is given on the y-axis. Separate boxes are provided for non-responder samples (n=3) and responder samples (n=3). (E) STRING network plot of the genes in the transmembrane receptor protein kinase activity gene ontology. Nodes represent proteins and edges joining nodes represent protein–protein associations (known interactions; blue—curated data, pink—experimentally determined. Predicted interactions; green—gene neighbours. Other interaction; black—coexpression). Genes of interest are circled in green. (F) Correlation analysis between % of inhibition of OC differentiation and serum vascular endothelial growth factor (VEGF) concentration.

Given the observed altered epigenetic landscape in RA MOs and the strong evidence that TNF drives joint destruction in RA, we hypothesised that the homoeostatic effect of TNF on circulating myeloid cells would be perturbed in RA and could thus promote OC-mediated erosive pathology. To assess the impact of this on responsiveness to TNF, CD14^+^ pre-OCs from RA and healthy controls were differentiated in the presence of RANK-L±TNF. While TNF consistently inhibited osteoclastogenesis in healthy controls, we observed a significant decrease in the capacity of TNF to inhibit osteoclastogenesis in patients with RA, with a certain degree of variance (figure 6A). In particular, 44% manifest TNF-mediated inhibition (responders), whereas in 56% of patients TNF was unable to inhibit osteoclastogenesis (non-responders; figure 6A, B). Remarkably, TNF had an enhancing pro-osteoclastogenic effect in 45% of the non-responder group. To further investigate the heterogeneity in the patients with RA, ChIPseq data of those patients that showed TNF-mediated inhibition (responders) and those that did not (non-responders) were compared. This analysis revealed that there were 4172 significantly differential peaks (figure 6C). Pathway enrichment showed that abundant peaks in RA MOs of the non-responder group were associated with transmembrane receptor protein kinase pathways and DNA-binding transcription repressor activity pathways (figure 6D and online supplemental dataset 2). Among the transmembrane receptor protein kinase pathways, CSF1R was found to be lower in the non-responders compared with the responders. STRING analysis of the identified transmembrane receptor protein kinase pathway associated genes also provided a link between CSF1R and NRP1 (figure 6E). Interestingly, NRP1 is known to act as a coreceptor for vascular endothelial growth factor (VEGF)-R^40^ in MOs/macrophage, and VEGFR expression and VEGF-mediated signalling has been associated with MO differentiation into OCs.^41 42^ Evaluation of RA patient serum revealed that high levels of VEGF correlate with response to TNF (figure 6F). Combined, these data suggest that in a proportion of patients with RA the high levels of VEGF correlate with a perturbation in the myeloid compartment, such that TNF homoeostatic control is diminished, thereby enhancing the potential for maturation of OCs derived from the circulating MO pool when they enter the joint.

10.1136/annrheumdis-2020-219262.supp3 Supplementary data

## Discussion

Our data reveal an unexpected role for TNF in the control of cell fate decisions in the myeloid compartment that regulates OC development and subsequent activation. In homoeostatic conditions, TNF can directly over-ride OC differentiation signals to which circulating myeloid precursors would otherwise respond. Furthermore, we have identified a specific human Lin^−^HLA^−^DR^+^CD14^−^CD16^−^CD11c^+^ precursor population that preferentially differentiates down the OC lineage, and that, based on its epigenetic state, is unresponsive to homoeostatic regulation imparted by TNF. In RA, a proportion of patients with moderate/severe disease activity exhibit failure of this regulatory role of TNF with some patients even exhibiting accelerated osteoclastogenesis on TNF exposure at this very early time point in the precursor differentiation pathway. This perturbed response is due to an altered MO epigenetic landscape. Taken together, our findings provide a novel, previously unrecognised hierarchy between TNF cytokine family members regulating cell fate decisions in both health and disease, which is mediated via the epigenetic set-point of circulating precursors defining their capacity to respond to cytokine combinations in the local milieu.

Our finding that homoeostatic TNF can control MO, but not other myeloid precursor differentiation in osteoclastogenic rich environments defines a paradigm in which priming of cells within the circulating myeloid compartment creates either a permissive or non-permissive epigenetic state that allows cells to differentiate down the most appropriate lineage. It is however, currently unclear how this epigenetic state is achieved in circulating MOs or how TNF drives additional changes, and thus the role of specific histone methyltransferase and/or demethylases should be evaluated in future studies.^43^ Previous studies have demonstrated that CD14^+^ MO-derived immature DC-like cells have the ability to transdifferentiate into OCs more efficiently than CD14^+^ MO progenitors.^19 44^ Note however, that circulating pre-DCs are the only cell subset capable of generating classical DCs while CD14^+^ MO can only generate DC-like cells.^7^ Our findings provide the first evidence that circulating human Lin^−^HLA^−^DR^+^CD14^−^CD16^−^CD11c^+^, which can also phenotypically be associated with the pre-DC population, can differentiate down the OC lineage. Whether Lin^−^HLA^−^DR^+^CD14^−^CD16^−^CD11c^+^/pre-DCs are the missing OC precursor in humans remains to be conclusively determined.

Based on this work, we suggest that in a normal self-resolving inflammatory event (which can include sterile/acute inflammation), Lin^−^HLA^−^DR^+^CD14^−^CD16^−^CD11c^+^ cells recruited to bone will still respond to the cytokine rich local environment by differentiating down the OC lineage and thus help maintain skeletal health. In contrast, circulating CD14^+^ MOs are in a non-permissive state and are inhibited from contributing to the OC pool. We recognise the limitations of our study; in that we have used in vitro methods to evaluate cell fate decisions within the myeloid compartment. Evidence to further support this theory would require human in vivo/ex vivo cell fate mapping studies to determine which precursors (Lin^−^HLA^−^DR^+^CD14^−^CD16^−^CD11c^+^ or CD14^+^ MOs) contribute to the bone-associated OC pool. Unfortunately, these assays are not currently possible. Further studies will need to define a way of identifying Lin^−^HLA^−^DR^+^CD14^−^CD16^−^CD11c^+^—versus MO-derived OCs in situ to better understand this alternative route to mature OCs. Moreover, they should also investigate the global molecular and epigenetic signature of Lin^−^HLA^−^DR^+^CD14^−^CD16^−^CD11c^+^ and CD14^+^ precursors cells, and their respective mature OCs, to ultimately identify the specific precursor cells and molecular mechanisms involved in inflammatory joint destruction.

Anti-TNF therapy is one of the gold standard treatments for multiple inflammatory-mediated diseases including RA. It is paradoxical however, given that TNF inhibitors are known to be antierosive,^45^ that we show an antierosive effect for TNF itself via a homoeostatic pathway in health. Crucially, we do show that in a proportion of patients with RA with have higher levels of VEGF, there is a failure in this homoeostatic pathway. Leading us to hypothesise that such failure may represent a route MO preconditioning and subsequent accelerated erosion in a subset of patients. Characterising the nature of such a failure may offer future therapeutic opportunities. For instance, determining how to re-engage this regulatory pathway may lead to new antierosive therapeutics that have the ability to re-sensitise some patients with RA to the regulatory element of TNF biology. This could also have the added value of reducing the capacity of TNF, produced for example during RA synovitis, to sensitise early precursors down the OC pathway. Finally, the newly identified Lin^−^HLA^−^DR^+^CD14^−^CD16^−^CD11c^+^ precursor route to mature OCs represents an unexplored independent mechanism to mature OCs and potentially erosive progression in RA. A detailed understanding of this new pathway could reveal an as yet untargeted pathway in the disease context. In summary, these data support further evaluation of these pathways in RA and other diseases associated with bone pathology to discover their utility as alternative therapeutic strategies to abrogate erosive progression.

## Data Availability

Data are available in a public, open access repository. Data and materials availability: ChIP-seq datasets were deposited in Gene Expression Omnibus (GEO) with the accession number ID GSE15291 and token irctaigmjlmxtkj (https://www.ncbi.nlm.nih.gov/geo/query/acc.cgi?acc=GSE152912)
