# Supplementary material for: TNF is a homoeostatic regulator of distinct epigenetically primed human osteoclast precursors
Source: Ann Rheum Dis. 2021 Mar 10;80(6):748–57. doi: 10.1136/annrheumdis-2020-219262 (PMC8142443; doi:10.1136/annrheumdis-2020-219262)
Supplement: Supplementary data [file annrheumdis-2020-219262supp001.pdf]

Online Supplementary Materials

TNF is a homeostatic regulator of distinct epigenetically primed human osteoclast precursors

Authors: Cecilia Ansalone, John Cole, Sabarinadh Chilaka, Flavia Sunzini, Shatakshi Sood, Jamie Robertson, Stefan Siebert, Iain B. McInnes, and Carl S. Goodyear.

Contents

- Pgs 2-13: Supplementary materials and methods.
- Pg 14: Figure S1. TNF-driven inhibition of osteoclastogenesis drives $CD14^+$ pre-cursors toward an intermediate Mϕ phenotype.
- Pg. 15: Figure S2. $CD11c^+$ pre-OCs produces IFNγ under TNF stimulation while $CD14^+$ pre-OCs produce pro-inflammatory cytokines.
- Pg 16: Figure S3. Etanercept restores osteoclasts differentiation in presence of TNF.
- Pg 17: Figure S4. TNF inhibition of osteoclast differentiation of $CD14^+$ pre-cursors is time dependant and delayed addition enhances osteoclastogenesis.
- Pg 18: Figure S5. TNF does not affect CSF1R expression.
- Pg 19: Figure S6. TNF does not affect cell apoptosis.
- Pg 20-21: Figure S7. TNFR2 expression increases during osteoclast differentiation and mediates TNF pro-osteoclastogenic effects in pre-fusion OCs.
- Pg 22: Figure S8. Comparison between RA blood and synovial CD1C cells.

Ansalone C, *et al. Ann Rheum Dis* 2021; 80:748–757. doi: 10.1136/annrheumdis-2020-219262

- Pg 23: Table S1. RA patient's characteristics. Table S2. Primer sequences used for quantitative RT-PCR. Table S3. Primer sequences used for ChIP-PCR of promoter regions of selected genes. References.

Supplementary materials and methods

Blood collection and cell isolation

Blood from healthy individuals and RA patients was collected in lithium heparin vacuum blood tubes (BD Vacutainer LH, 170 IU). For certain RA patients, blood for serum separation was also collected (BD Vacutainer SST II Advance). Blood samples from patients diagnosed with RA (with a diagnosis meeting the 2010 ACR/EULAR RA criteria) were collected at Rheumatology clinics (Glasgow, UK); all patients were naïve to TNF-biologics and had moderate to severe disease based on their Disease Activity Score (DAS28). Table S1 summarizes the characteristics of our study population. The study protocol was approved by the West of Scotland Research Ethical Committee (11/S0704/7). All the donors provided signed informed consent. Alternatively, buffy coat was obtained from the Scottish National Blood Transfusion Service (approved by Glasgow NHS Trust-East Ethics Committee). Peripheral blood mononuclear cells (PBMCs) were extracted by density gradient separation using Ficoll-paque PLUS (GE Healthcare Life Science). $CD14^+$ monocytes and $CD11c^+$ precursors were magnetically enriched from PBMCs using EasySep™ Human CD14 Positive Selection Kit and EasySep™ Human Myeloid DC Enrichment Kit (STEMCELL Technologies) respectively. Purity was assessed via flow cytometry staining and showed purity≥96%.

Cell cultures and osteoclast differentiation and analysis

Freshly isolated PBMCs, magnetically enriched $CD14^+$ monocytes and $CD11c^+$ precursors (purity≥96%), as well as fluorescently sorted populations (purity≥99%), were re-suspended at $1x10^6$/ ml in complete α-MEM medium (supplemented with 10% of heat inactivated foetal bovine serum (FBS), 0.02 mML-glutamine, 10 units/ml penicillin, 0.1

Ansalone C, *et al. Ann Rheum Dis* 2021; 80:748–757. doi: 10.1136/annrheumdis-2020-219262

mg/ml streptomycin) (Invitrogen, Thermo Fisher Scientific), plated at density of 1x10^5/well in 96-well plates either on plastic or on mineral-coated plates (Corning osteo-assay surface microplate) and stimulated with 25 ng/ml macrophage–colony-stimulating factor (M-CSF; Peprotech). After overnight incubation cells were defined as $CD14^+$ pre-osteoclasts (pre-OCs) and $CD11c^+$ pre-OCs (approximately 18h) and used for down-stream applications. Osteoclasts were differentiated by stimulating pre-OCs with 25ng/ml (unless where otherwise stated) receptor activator of nuclear factor kappa-B ligand (RANK-L), alongside 25ng/ml M-CSF. Tumor necrosis factor alpha (TNF) was used at 0.1, 1, and 10 ng/ml and added at different time points during osteoclastogenesis, as specified in figure legends. Medium was refreshed every 3-4 days. For cultures on plastic, osteoclast differentiation was assessed by fixation of cells and staining with tartrate-resistant acid phosphatase (TRAP) kit (Sigma-Aldrich), in accordance with the manufacturer's instructions. For the resorption assay, cells were removed from mineral-coated plates using a 10-15% sodium hypochlorite solution (Sigma-Aldrich) and the mineral substrate left to air dry. Reconstructed digital images of the entire well were acquired using an EVOS FL Auto Cell Imaging System (Life Technologies). Osteoclasts were identified as $TRAP^+$ multinucleated (nuclei≥3) cells (MNCs) and counted using Fiji software (ImageJ). Resorption was calculated using Fiji software (ImageJ) by converting the images into 8-bit and setting the threshold at 223 to 254; resorption areas were calculated as % of the total area of the well.

Signalling inhibition and TNF receptor blockade during osteoclastogenesis

TNF receptor fusion protein etanercept (Enbrel, Amgen) was added to osteoclast cultures at 1, 10, or 50 μg/ml alongside with TNF. Additionally, purified antibody specifically recognizing TNF receptor 1 (mouse anti-human CD120a; αTNFR1; eBioscience) and TNF receptor 2 (rat anti- human CD120b; αTNFR2; BioLegend) were added to

osteoclast cultures in the presence of RANK-L ± TNF. Appropriate isotype antibody controls were purchased from BioLegend and used as negative controls. All antibodies and isotypes were used at 10 µg/ml. In some experiments, TPCA-1 ([5-(p-Fluorophenyl)-2-ureido]thiophene-3-carboxamide; Sigma-Aldrich) was used to specifically inhibit IκB kinase-2 (IKK-2; IC50 = 17.9 nM). TPCA-1 was added at 100 and 300nM at the beginning of the osteoclast culture alongside 25 ng/ml RANK-L ± 10 ng/ml TNF. After 24h the inhibitor was washed off and medium replaced with 25 ng/ml RANK-L ± 10 ng/ml TNF. 0.06% Dimethyl sulfoxide (DMSO) was used as vehicle control.

Cell preparation for flow cytometry applications

Freshly isolated PBMCs were suspended in DPBS supplemented with 1% FBS, 0.1% NaN_3 and 5mM EDTA and stained for flow cytometry. Alternatively, freshly enriched $CD14^+$ monocytes were incubated overnight with 25ng/ml M-CSF to generate $CD14^+$ pre-OCs (0h) and then stimulated with 25 ng/ml RANK-L ± 10 ng/ml TNF for 72h. Control wells received M-CSF alone. Cell were taken at 0 and 72h and stained for flow cytometry. To sort specific populations, PBMCs were stained with flow cytometry antibodies in sterile DPBS supplemented with 1% FBS and 2mM EDTA and sorted using an BD FACSAria III cell sorter with an 85µm nozzle (BD Bioscience). Cells were sorted into tubes containing complete α-MEM, re-suspended at $1x10^6$ cells/ml and incubated overnight with 25ng/ml M-CSF for downstream osteoclast cultures. Post-sorting check assessed purity≥99%. Antibody staining was performed in the dark for 15 minutes at 4˚C. Additional incubation for 20 minutes at 4˚C with PerCP/Cy5.5 Streptavidin (BioLegend) was performed where required. Washed cells were acquired with an LSR II cytometer (BD Bioscience) and data analysed with a Flowjo 10.0.5 software (Tree Star).

Ansalone C, *et al. Ann Rheum Dis* 2021; 80:748–757. doi: 10.1136/annrheumdis-2020-219262

Antibodies used for flow cytometry

Anti-human antibodies used for flow cytometry applications are listed below: APC-Cy7 CD14 (M5E2; BioLegend), PE-CF594 mouse anti-human CD14 (MφP9; BD Bioscience), V500 CD16 (3G8; BD Biosciences), PE-Cy7 HLA-DR (G46-6; BD Biosciences); Alexa Fluor-700 CD11c (B-ly6; BD Bioscience), Brilliant Violet-605 CD123 (6H6; BioLegend), biotin TNFR1/CD120a (MABTNFR1-B1; BD Bioscience), Alexa Fluor-647 TNFR2/CD120b (hTNFR-M1; BD Bioscience), PE CD3 (UCHT1; BD Bioscience), PE CD19 (J3-119; Beckman Coulter), PE CD56 (MY31; BD Biosciences); PE CD15 (VIMC6; Miltenyi Biotec); APC CD80 (2D10; BioLegend); FITC CD64 (10.1; BioLegend); Brilliant Violet 421 CD206 (15-2; BioLegend). Mouse IgG2a (G155-178; BD Bioscience) and rat IgG2a (A95-18; BD Biosciences) were used as isotype controls for TNFR1 and TNFR2 respectively.

Labelling of RANK-L and fluorescent protein up-take

Recombinant human soluble RANK-L (Peprotech) was re-suspended at 1 mg/ml in dH_2O and labelled with Pacific Blue™ protein labelling kit, following the manufacturer's instructions (Thermo Fisher Scientific). Concentration of the labelled cytokine (RANK-L^{PB}) was assessed by Nanodrop and adjusted to 100μg/ml in 0.1% bovine serum albumin (BSA) in Dulbecco's phosphate-buffered saline (DPBS; Life Technologies, Thermo Fisher Scientific).

CD14^{+} monocytes were differentiated into OCs for 72h in the presence of 25 ng/ml RANK-L ± 10 ng/ml TNF and then incubated at 37˚C for 1 hour with 100ng/1x10^{6} cells RANK-L^{PB} in complete α-MEM medium (no FBS). Medium alone was used as negative control. After the incubation, cells were washed and re-suspended in DPBS supplemented

Ansalone C, *et al. Ann Rheum Dis* 2021; 80:748–757. doi: 10.1136/annrheumdis-2020-219262

with 1% FBS, 0.1% NaN_3 and 5mM Ethylene-di-amine-tetra-acetic acid (EDTA) for flow cytometry analysis.

Cytokine production analysis

CD14$^+$ monocytes, after overnight incubation, were stimulated with different combinations of 25ng/ml M-CSF, 25ng/ml RANKL, and 10ng/ml TNF. Granulocyte macrophage colony-stimulating factor (GM-CSF; Peprotech) was used at 100 ng/ml. After 6 days medium was removed and replaced with media containing vehicle control or 100ng/ml lipopolysaccharide (LPS from Salmonella Minnesota R595; InvivoGen). After 18h supernatants were stored, and cytokine production was assessed. Alternatively, CD14$^+$ monocytes and CD11c$^+$ precursors were magnetically enriched, incubated overnight with 25ng/ml M-CSF to generate pre-OCs and then stimulated for 72h with 25ng/ml RANKL ± 10ng/ml TNF. Supernatants were collected and cytokine concentration assessed using the Meso Scale Discovery technology (Meso Scale Diagnostics). Specifically, a V-PLEX Pro-inflammatory Panel 1 Human Kit (Meso Scale Diagnostics) was used to determine concentrations of IL-10, IL-12p70, IL-1β, IL-6, and IFNγ in cell supernatants, following manufacturer's instructions. Analysis was performed using the MSD Discovery Workbench analysis software (Meso Scale Diagnostics).

RNA isolation and quantitative RT-PCR

Cells were lysed in RLT buffer (Qiagen) containing 1% beta-mercaptoethanol. mRNA was extracted according to the manufacturer's instructions using the RNeasy Micro Kit (Qiagen) and eluted in 15 µl RNAse-free H_2O. cDNA was synthesized using High capacity cDNA reverse transcription kit (Applied Biosystems, Thermo Fisher Scientific). A

quantity of 1 ng cDNA was taken for RT-qPCR analysis using Power SYBR Green PCR Master Mix (Applied Biosystems, Thermo Fisher Scientific), and a QuantStudio 6 machine (Thermo Fisher Scientific). A quantity of 1 ng cDNA was taken for RT-qPCR analysis using Power SYBR Green PCR Master Mix (Applied Biosystems, Thermo Fisher Scientific), and a QuantStudio 6 machine (Thermo Fisher Scientific). Relative gene expression and fold change was calculated using the comparative C_T method[1]. ΔC_T values were calculated as $C_{T\ gene\ of\ interest} - C_{T\ housekeeping\ gene}$ for each sample. The ΔC_T is then converted to linear relative gene expression using the following formula $2^{-\Delta CT}$. Fold change was measured as $2^{-\Delta\Delta CT}$, where $\Delta\Delta C_T$ corresponded to $\Delta C_{T\ control\ sample} - \Delta C_{T\ treated\ sample}$. Oligonucleotides were designed in house and listed in Table S2. Primers for RANK and GAPDH were designed on exon span junctions. In order to avoid genomic contamination, endogenous DNA was digested using RNase-Free DNase set during mRNA extraction, as described in the manufacturer's instructions (Qiagen).

Chromatin Immunoprecipitation (ChIP)

Cells were fixed in 1% formaldehyde for 10 minutes at room temperature, followed by quenching with 125mM Glycine for 5 minutes. Cells were scraped and collected by centrifugation at 4˚C. Pelleted cells were washed twice with cold DPBS (GIBCO, Thermo Fisher Scientific) and lysed in lysis buffer (20mM Hepes pH 7.6, 1% SDS, 1X Protease Inhibitor Cocktail and 10Mm Sodium butyrate). Chromatin samples were sonicated for 14±2 cycles of 30 sec ON/30 sec OFF with the Bioruptor Pico sonication device (Diagenode) until most of the DNA fragments were 100-600 bp long (average length 200 bp). The sonicated samples were then centrifuged at ≥13000 rpm for 5 minutes at 4˚C to collect the supernatant containing the soluble chromatin fraction.

Ansalone C, *et al. Ann Rheum Dis* 2021; 80:748–757. doi: 10.1136/annrheumdis-2020-219262

For each IP, 20µl of Dynabeads Protein A (Invitrogen, Thermo Fisher Scientific) were used. For antibody conjugation, beads were washed in ChIP dilution buffer (1% Triton x-100, 1.2 mM EDTA, 16.7mM Tris buffer pH 8, and 167mM NaCl) containing 0.01% SDS and 0.1% BSA and incubated with H3K4me3 antibody (Merk Millipore (1.5ug/IP)) in the same buffer for 1hr at room temperature with rotation. After conjugation, beads were washed, and the chromatin added; conjugated beads and chromatin were incubated in ChIP dilution buffer on a rotator for 3h at 4˚C. After incubation, beads were washed once with ChIP washing solution 1 (2mM EDTA, 20mM Tris buffer (pH 8), 1% Triton x-100, 0.1% SDS, and 150mM NaCl), twice with ChIP washing solution 2 (2mM EDTA, 20mM Tris buffer (pH 8), 1% Triton x-100, 0.1% SDS, and 500mM NaCl), and twice with ChIP washing solution 3 (1mM EDTA, 10mM Tris buffer (pH 8)). Finally, the beads were eluted in 100µl elution buffer (0.5% SDS, 300mM NaCl, 5mM EDTA, and 10mM Tris (pH 8)) containing 200µg/ml Proteinase K (Sigma-Aldrich). De-crosslinking was done by incubating samples at 55˚C for 1h followed by overnight at 65˚C. The supernatant containing the immunoprecipitated DNA was purified using Qiagen MiniElute PCR purification kit, following manufacturer instructions. Eluted DNA was used for qPCR and ChIP-seq applications. Gene promoter regions were obtained using the UCSC Genome Browser; primers were designed in house and listed in Table S3.

ChIP-seq data analysis

ChIP-seq libraries were prepared using the NEB NEXT Ultra II DNA-library prep kit (E7645S for ChiP and E7600S for input) and samples were sequenced on an Illumina Next-Seq to a mean depth of 38 million reads. The read length was 75pb SE. The read quality of ChIP-seq dataset was verified using fastQC (v0.11.7) with each sample showing a mean per base quality > 30 at all read positions. The data aligned to the human genome (GRCh38

Ansalone C, *et al. Ann Rheum Dis* 2021; 80:748–757. doi: 10.1136/annrheumdis-2020-219262

version 94) using bowtie 2 (v2.3.5) with default parameters for indexing and alignment. A mean alignment of 28 million uniquely mapping reads per sample (74%) was observed. Per sample wig files were generated using the PeakRanger (1.18) wig command with format bam. Bigwigs were generated from the wig files using UCSC tools wigToBigWig (v4), with chromosome sizes as determined by UCSC tools faSize. The per sample H3K4me3 peaks were called with macs2 (v2.1.1.20160309) callpeak using the input BAM file for each sample as the control, a genome size of 2,945,849,067bp and specifying --format BAM. The alignment and peak data were inspected on the IGV genome browser (v2.7.2). Two samples (RA2 and RA4) showed high levels of noise (observable as non-peak aligned reads), low numbers of reads at peaks (10x lower than the mean) and low technical correlation with other samples. These samples were therefore excluded from the downstream analysis. Next, differential peaks between the HC and RA samples were called using the R (v3.6.2) package DiffBind (v2.14.0) using the per sample MACs broad peaks as Peaks and the per sample input BAM files as bamControl. The model was set to HC vs RA. All other parameters were left to default. DiffBind identified 6,763 significantly differential peaks at < 5% FDR from a consensus set of 75,425 peaks. The DiffBind normalised peak intensities were used for the downstream heatmap and GO analysis. The 6,763 differential peaks were annotated using Homer (v4.11.1) annotatePeaks with the databases organism human (v6.3), promoters human (v5.5) and genome hg38 (v6.4). The Gene Ontology (GO) enrichment was calculated using Homer findMotifs.pl inputting the entrez ID of the nearest TSS (within 50kb) for each peak (from the annotated peaks file) as the candidate genes. All other settings were left to default. Enriched ontologies were identified as $p < 0.0001$ and (to reduce database redundancy) a term size > 5 and < 250. The GO enrichment results are provided in supplementary dataset 1.

To identify differential peaks between responders and non-responders, firstly, responder (R) samples were identified as having a percent of inhibition > 68% (RA3, RA7

and RA11) and non-responder NR) samples as < 25% (RA1, RA6 and RA10). Next differential peaks were identified using the methods as described above however with the model R vs NR. DiffBind identified 4,172 significantly differential peaks at < 5% FDR from a consensus set of 65,717 peaks. Differential peaks were annotated, and enriched GO identified as described above. The GO enrichment results are provided in supplementary dataset 2.

ChIP-seq visualization

The heatmap of the 6,763 differential peaks between HC and RA (figure 5A) was generated using the R library amap (v0.8-17). Rows were clustered using the function hclust with Spearman distances and mean reordering. Diffbind normalised peak intensities were row scaled into z-scores.

To generate the network of enriched GO (figure 5C) the Homer enrichment results for biological process, molecular function and cellular component were concatenated and filtered to include only terms with an enrichment value < 0.0001 and between 5 and 250 genes with significant peaks. Each remaining ontology was considered a node and edges were drawn between two nodes where at least 50% of the genes with significant peaks were in common (Szymkiewicz-Simpson coefficient) and there were at least 5 overlapping genes with significant peaks. The network was drawn using the R package ggnet2 (v2.4) under default settings. To highlight the major functional groups, clusters with fewer than 5 nodes were removed, and representative names were given.

To generate the candidate peak (RANK, TNFR1 and TNFR2) bar-plots (Figure 5C) the promoter consensus peak (as generated previously by Diffbind) for each gene was identified using IGV. Next the read count at each peak for each H3K4me3 and input sample

was determined using the Bedtools (v2.26) multicov function. The aligned library size for each sample was determined using Samtools (v1.7) view -c with -F 260. Next the counts per million (CPM) ((count / library size) x1,000,000) at each peak was determined for each H3K4me3 and input sample. Finally, the input normalised peak intensities were calculated as: H3K4me3 CPM – Input CPM.

To create STRING networks we used the dedicated website (https://string-db.org) and the multiple proteins function under default settings[2].

Comparison between blood CD1C and Classical Monocyte populations.

PBMC single cell RNA-seq dataset was obtained from GEO (GSE94820) as raw counts. These were then partitioned into the pre-identified CD1C and Classical Monocyte populations and differential expression performed using DESeq2. The data was then explored with Searchlight2 using an adjusted $p < 0.01$ and absolute log2 fold change >1 and the GO biological process database. All other settings were left to default.

RA serum analysis

Serum from RA patients was collected by centrifugation at 1200xG for 10' minutes, aliquoted and stored at -80˚C. Serum VEGF was evaluated using a U-PLEX Human VEGF-A (Meso Scale Diagnostics). Analysis was performed using the MSD Discovery Workbench analysis software.

Ansalone C, *et al. Ann Rheum Dis* 2021; 80:748–757. doi: 10.1136/annrheumdis-2020-219262

Statistical analysis

Prism 6 (Graphpad) was used to perform all statistical analysis and statistical tests used are indicated in the figure legends. P values less than or equal to 0.05 were considered significant.

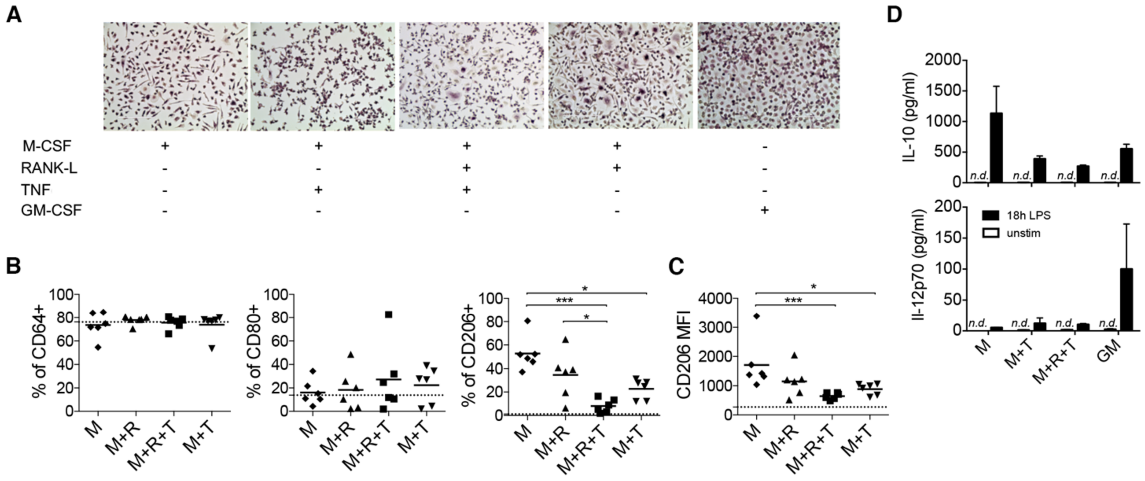

Figure S1. TNF-driven inhibition of osteoclastogenesis does not affect apoptosis and drives CD14⁺ pre-cursors toward an intermediate Mϕ phenotype. $CD14^+$-derived OC pre-cursors were cultured in the presence of 25ng/ml M-CSF (M) ± 25ng/ml RANK-L (R) and ± 10ng/ml TNF (T) in various combinations. **(A)** Representative 20X magnification of TRAP-stained cultures after 3 days of cytokine stimulation. **(B)** Percentage of single cells expressing CD64, CD80, and CD206 after 72h of cytokine stimulation. **(C)** MFI of CD206 after 72h of stimulation with M, M+R, M+T, M+R+T. **(B-C)** Dotted line shows baseline at 0h of $CD14^+$-derived OC precursors prior R±T stimulation. Bars show mean of n=6 of two experiments pooled together. Data were analysed with Friedman test of variance and Dunn's multiple comparisons test. ***$P \leq 0.001$; *$P \leq 0.05$. **(D)** IL-10 and IL-12 release after culturing the cells for 6 days with M, M+T, M+R+T, or 100 ng/ml GM-CSF (GM). Cytokine release was measured after replacing the media with complete medium ± 100 ng/ml LPS for 18h (unstim = no LPS; n.d.=non-detectable). Graphs show mean±SD of n=3.

Ansalone C, *et al. Ann Rheum Dis* 2021; 80:748–757. doi: 10.1136/annrheumdis-2020-219262

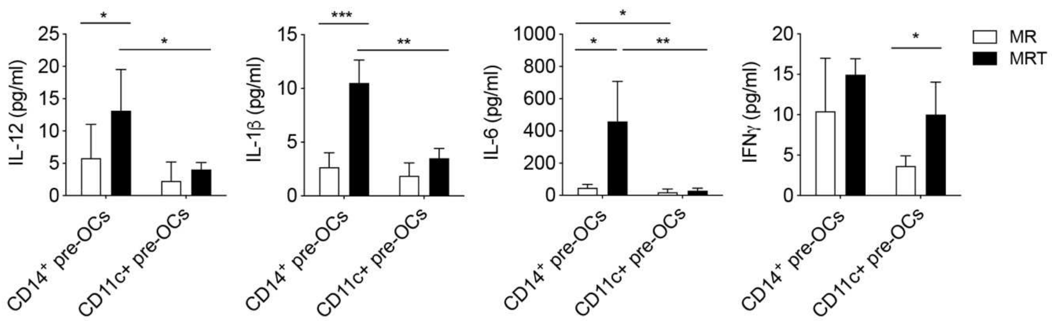

Figure S2. CD11c+ pre-OCs produces IFNγ under TNF stimulation while CD14+ pre-OCs produce pro-inflammatory cytokines. PBMCs were isolated and CD14+ monocytes (MOs) and CD11c+ precursors were magnetically enriched and incubated overnight with 25ng/ml M-CSF to generate CD14+ and CD11c+ pre-OCs, following by 72h RANK-L stimulation ± TNF (25ng/ml and 10ng/ml respectively). Cell supernatants were analysed for IL-12, IL-1β, IL-6, and IFNγ concentration. Bars show mean±SD of n=3-4. Statistical analysis was done using paired 2-way ANOVA and Sidak's multiple comparison tests.

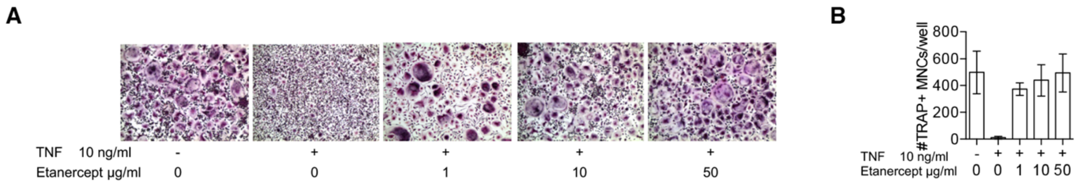

Figure S3. Etanercept restores osteoclasts differentiation in presence of TNF. $CD14^{+}$ monocytes were differentiated into OCs and OCs quantified as in Figure 1. **(A)** Representative 10X digital images of TRAP staining (purple) and **(B)** quantification of number of OCs per well after 6 days with 25 ng/ml RANK-L ± 10ng/ml TNF ± increasing concentration of etanercept (1, 10, or 50µg/ml). Bars = mean±SD (n=3). Data were analysed with two-way ANOVA and Tukey's multiple comparisons test. ****$P \leq 0.0001$.

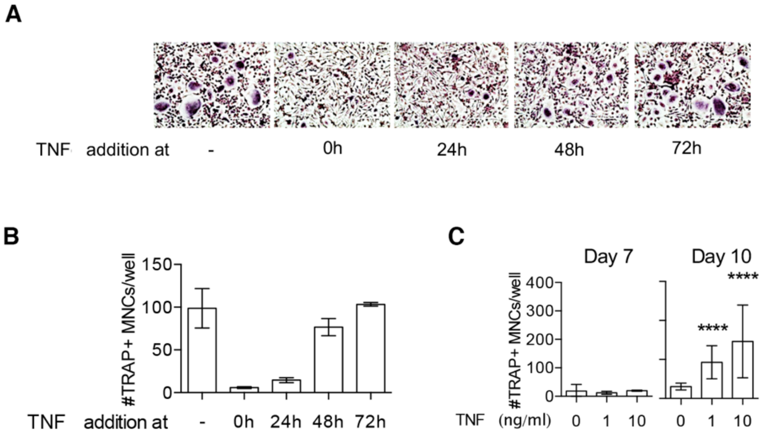

Figure S4. TNF inhibition of osteoclast differentiation of CD14$^+$ pre-cursors is time dependant and delayed addition enhances osteoclastogenesis. (A) Representative 20X digital images of TRAP staining and **(B)** quantification of TRAP+ MNCs/well differentiated from CD14$^+$ OC pre-cursors in the presence of 25ng/ml RANK-L for 6 days; 10ng/ml TNF was added onto the culture after 24, 48, or 72h. Error bars show mean ± SD of technical replicates of one representative experiment. The experiment was repeated twice, and similar results observed. **(C)** Quantification of TRAP+ MNCs per well after 6 days (at day 7 of the culture) or 9 days (at day 10 of the culture) of 72h of 1ng/ml RANK-L followed by additional stimulation with 1 or 10ng/ml TNF. Data were analysed using 2-way ANOVA and Dunnett's post-hoc test, comparing all data to 0 ng/ml (n=3); ****P<0.0001.

Ansalone C, *et al. Ann Rheum Dis* 2021; 80:748–757. doi: 10.1136/annrheumdis-2020-219262

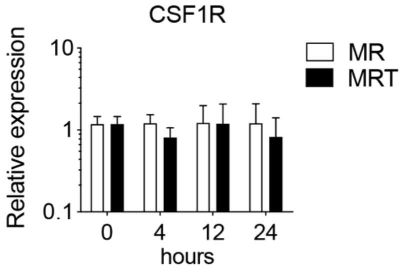

Figure S5. TNF does not affect CSF1R expression. Enriched CD14+ monocytes (MOs) were incubated overnight with 25ng/ml to generate CD14+ pre-OCs. CD14+ pre-OCs were then differentiated in the presence of 25ng/ml M-CSF + 25ng/ml RANK-L (MR) or MR +10ng/ml TNF (MRT). mRNA expression of CSFR1 was evaluated at 0, 4, 12, and 24h after cytokine addition on CD14+ pre-OCs. n=4.

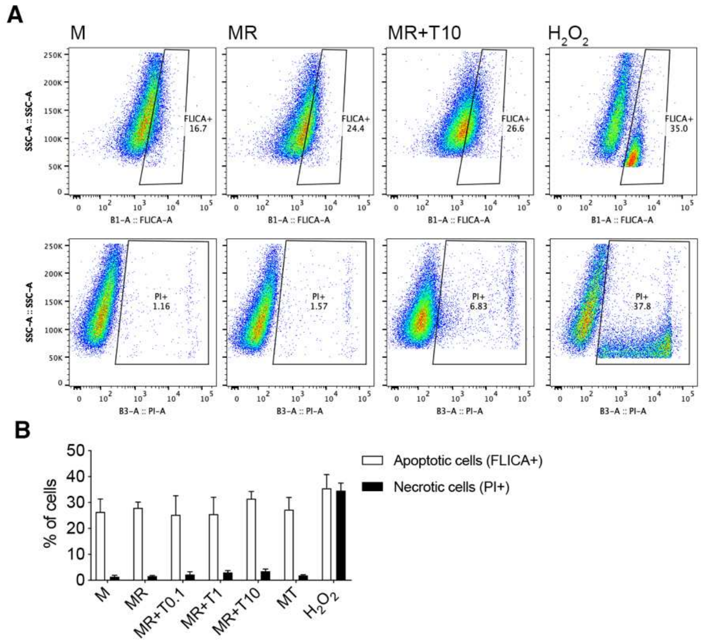

Figure S6. TNF does not affect cell apoptosis. CD14+ monocytes were enriched from PBMCs and incubated overnight with 25 ng/ml M-CSF to obtain CD14+-derived OC precursors (time 0h); these cells were subsequently incubated with 25ng/ml M-CSF ±25ng/ml RANK-L (MR) ±TNF at different concentrations (0.1, 1, or 10 ng/ml; T0.1, T1 and T10 respectively) for 48h and viability quantified using the Vybrant ® FAM Poly Caspases Assay Kit (ThermoFisher Scientific) following the manufacturer's instructions. H_2O_2 was used as positive control. **(A)** Representative density plots showing gating strategy for calculating apoptotic cells (FLICA +) and necrotic cells (PI+) in M-CSF (M), M±RANK-L (MR), MR±10ng/ml TNF (MR+T10), and H_2O_2 samples. **(B)** Quantification of % of apoptotic cells (FLICA +) and necrotic cells (PI+) in n=3 independent experiments. Error bars show mean±SD.

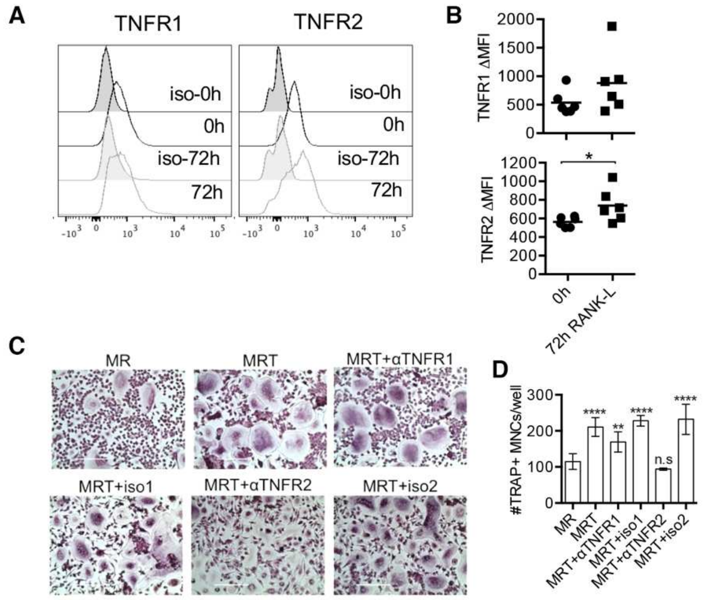

Figure S7. TNFR2 expression increases during osteoclast differentiation and mediates TNF pro-osteoclastogenic effects in pre-fusion OCs. (A-B) $CD14^+$ monocytes were enriched from PBMCs and incubated overnight with 25 ng/ml M-CSF to obtain $CD14^+$-derived OC precursors (time 0h); these cells were subsequently incubated with M-CSF and RANK-L (MR) for 72h to differentiate into pre-fusion OCs. **(A)** Representative half-offset histograms show TNFR1 and TNFR2 fluorescence of $CD14^+$-derived OC precursors (0h) and after 72h with 25ng/ml RANK-L (iso=isotype control for each of the TNFR antibody). **(B)** Graphs show ΔMFI of TNFR1 and TNFR2 of total single live cells at 0h and 72h of 25ng/ml RANK-L. ΔMFI of TNFR1 and TNFR2 was calculated by subtracting the MFI of the TNFR to the relative MFI of the isotype control. Data were analysed using Wilcoxon rank test for paired data. **P*≤0.05. n=6 from 2 different experiments pooled together. **(C-D)** $CD14^+$-derived OC precursors were differentiated with 1ng/ml RANK-L (MR) for 72h into pre-fusion OCs and then 10ng/ml TNF was added onto the culture (MRT) ± antibody blocking TNFR1 or TNFR2 (αTNFR1 and αTNFR1) or ± the respective isotype controls (iso1 and iso2 respectively). **(C)** Representative 20X digital images of TRAP staining at day 10 **(D)**

Ansalone C, *et al. Ann Rheum Dis* 2021; 80:748–757. doi: 10.1136/annrheumdis-2020-219262

quantification of numbers of OCs per well. Statistical significance was assessed with 2-way ANOVA and Sidak's multiple comparisons test, comparing all data to MRT. Error bars = mean±SD of n=3. ****P<0.0001.

Ansalone C, *et al. Ann Rheum Dis* 2021; 80:748–757. doi: 10.1136/annrheumdis-2020-219262

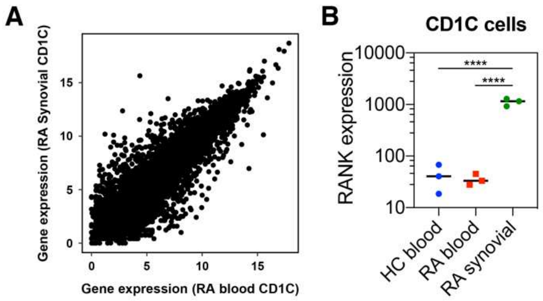

Figure S8 - Comparison between RA blood and synovial CD1C cells. (A) Comparison of global gene expression profiles between RA patient blood and synovium. Each dot is a gene. The x and y axis show expression (log10) of each gene in RA patient blood and synovium respectively. **(B)** RANK gene expression in CD1C cells isolated from HC blood, RA blood and RA synovial. Data were analysed with one-way ANOVA and Holm-Sidak's multiple comparisons test. ****=P<0.0001 and n=3.

Age	67.54±9.04
Disease duration (yrs)	11.5±8.61
Seropositive	7(12)
CDAI	18.04±6.96
SDAI	24.69±13.16
DAS-28(ESR)	4.42±0.87
DAS-28(CRP)	4.14±0.82

Table S1. RA patient's characteristics. CDAI = Clinical Disease Activity Index; SDAI = Simplified Disease Activity Index; DAS = Disease Activity Score; ESR = Erythrocyte Sedimentation rate; CRP= C-reactive Protein. Values are expressed as mean±SD.

RANK Forward	GCTGTAACAAATGTGAACCAGGA
RANK Reverse	GCCTTGCCTGTATCACAAACT
CFS1R Forward	TCCCAGTGATAGAGCCCAGT
CFS1R Reverse	CAGGGTCCAGTGAGGTGATG
GAPDH Forward	GAAGGACTCATGACCACAGT
GAPDH Reverse	GTAGAGGCAGGGATGATGTT

Table S2. Primer sequences used for quantitative RT-PCR.

RANK promoter Forward	GCTGGCCCCAACATTTTGAA
RANK promoter Reverse	CGTCGAGAATGAACGGGAGG
CSF1R promoter Forward	ACACTGGACACACGTTCCTC
CSF1R promoter Reverse	TCCAAACTCTGTGGTTGCCT
GAPDH promoter Reverse	TCATCCAAGCGTGTAAGGGT
GAPDH promoter Forward	ACTGAGATTGGCCCGATGG

Table S3. Primer sequences used for ChIP-PCR of promoter regions of selected genes.

References

1 Schmittgen TD, Livak KJ. Analyzing real-time PCR data by the comparative CT method. *Nat Protoc* 2008;**3**:1101–8. doi:10.1038/nprot.2008.73

2 Szklarczyk D, Gable AL, Lyon D, *et al.* STRING v11: Protein-protein association networks with increased coverage, supporting functional discovery in genome-wide experimental datasets. *Nucleic Acids Res* 2019;**47**:D607–13. doi:10.1093/nar/gky1131
